# Explaining the “Unexpectedly High” Frequency of Invasive Fungal Disease in a Cohort Study of Solid Organ Transplant Recipients Treated With Belatacept

**DOI:** 10.1093/ofid/ofae291

**Published:** 2024-05-20

**Authors:** Madeleine R Heldman, Barbara D Alexander, John R Perfect, Ilan S Schwartz, Eileen K Maziarz

**Affiliations:** Division of Infectious Diseases, Department of Medicine, Duke University School of Medicine, Durham, North Carolina, USA; Division of Infectious Diseases, Department of Medicine, Duke University School of Medicine, Durham, North Carolina, USA; Division of Infectious Diseases, Department of Medicine, Duke University School of Medicine, Durham, North Carolina, USA; Division of Infectious Diseases, Department of Medicine, Duke University School of Medicine, Durham, North Carolina, USA; Division of Infectious Diseases, Department of Medicine, Duke University School of Medicine, Durham, North Carolina, USA

To the Editor—We took great interest in Bell et al.'s retrospective cohort study of invasive fungal disease (IFD) in solid organ transplant (SOT) recipients treated with belatacept [1]. The authors conclude that IFD incidence was “unexpectedly high”: 33 of 215 (15%) of SOT recipients and 23 of 56 (41%) of lung recipients developed at least 1 IFD. Although belatacept and concomitant immunosuppression may plausibly increase IFD risk, we are concerned that lack of standardized IFD definitions in this study overestimates IFD frequency and confounds comparisons to other cohorts in the literature.

Because fungi often colonize nonsterile sites and clinical and radiographic findings of IFD can be nonspecific, it is important to employ standardized definitions when studying IFD. The European Organization for the Research and Treatment of Cancer and the Mycoses Study Group Education and Research Consortium (EORTC/MSGERC) have created standardized research definitions of IFD and categorize cases as proven, probable, or possible based on diagnostic certainty. The term “possible IFD” encompasses scenarios in immunocompromised hosts in which clinical features of IFD are present but supportive mycologic findings are absent [2, 3]. Because of the limitations and confusion surrounding the concept of possible IFD in research settings, the most recent revisions of EORTC/MSGERC consensus criteria, published in 2020, diminish the category of possible IFD [4]. Additionally, the most robust multicenter studies describing IFD epidemiology in SOT recipients focus exclusively on proven and probable IFD [5–7]. For example, a landmark study from the Transplant Associated Infection Surveillance Network, which is cited by the authors to estimate IFD incidence in belatacept-unexposed SOT recipients, includes cases of proven or probable IFD only [5]. Although it is unclear which specific version of the EORTC/MSGERC definitions were used by Bell et al, 10/36 (28%) cases were classified as possible IFD. Exclusion of cases classified as possible IFD would facilitate smoother comparisons to external SOT recipient populations.

Differentiating fungal airway colonization from invasive pulmonary or tracheobronchial infection is particularly important when studying IFD in lung transplant recipients (LTRs). Surveillance studies demonstrate that 25%–30% of all LTRs develop airway colonization with *Aspergillus* or other molds, but invasive pulmonary disease or tracheobronchitis occurs in fewer than one third of colonized patients [8, 9]. It is important to note that antifungals are commonly initiated for *Aspergillus* colonization in LTRs, and such cases may be coded in electronic medical records as “pulmonary aspergillosis” or “fungal pneumonia,” even when research definitions of IFD are not fulfilled. Although isolation of *Aspergillus* from bronchoalveolar lavage fluid in LTRs meets the mycological and host requirements for probable IFD, specific clinical features must also be present to completely fulfill the 2008 and 2020 EORTC/MSGERC definitions of probable pulmonary aspergillosis or tracheobronchitis [2, 4]. The frequency of pulmonary aspergillosis in LTRs reported by Bell et al. was remarkably close to the expected frequency of *Aspergillus* colonization: 17/56 (30%) LTRs had aspergillosis, and the majority of aspergillosis cases (18/20, 90%) were pulmonary infections. Criteria used to establish cases of pulmonary *Aspergillus* infections are not included in Bell et al's report, and readers cannot be certain that such cases do not reflect airway colonization.

The study's classification of *Candida* infections may further conflate reported IFD frequency. Three of the 8 reported cases of candidiasis were cases of *Candida* esophagitis. There are no specific criteria for possible or probable *Candida* esophagitis in the 2020 or earlier EORTC/MSERGC criteria [2–4]. The esophagus is not a normally sterile site and only histological evidence of fungal invasion can fulfill criteria for proven IFD. The authors should clarify whether these 3 cases of *Candida* esophagitis were biopsy proven.

Bell et al do not specify follow-up durations, and the reported IFD “incidence” may actually reflect lifetime IFD prevalence. IFD epidemiology is often measured as either the cumulative incidence of first IFD (proportion of a population with at least 1 IFD over a certain period) or incidence rate (number of new IFD cases per person-time at risk). For example, the Transplant Associated Infection Surveillance Network reported the cumulative incidence of IFD within 1 year after transplantation [5]. Examining cumulative incidence over a specific timeframe (eg, 1 year after transplantation) or calculating incidence rate would facilitate comparisons to external cohorts.

We commend Bell et al for their effort in investigating fungal infections in SOT recipients treated with belatacept. However, further details are necessary to contextualize reported data and make accurate comparisons to external cohorts. We wholeheartedly agree with the authors that further research is required to clarify the IFD risks with belatacept and other new immunosuppressive agents.

## Notes

***Financial support.*** None.
